# The social and cultural meanings of infertility for men and women in Zambia: legacy, family and divine intervention

**Published:** 2020-10-08

**Authors:** S Howe, JM Zulu, J Boivin, T Gerrits

**Affiliations:** Department of Anthropology, University of Amsterdam, Postbus 15509, 1001 NA Amsterdam, the Netherlands; School of Public Health, University of Zambia (Ridgeway Campus), P.O Box 50110, Lusaka, Zambia; School of Psychology, Cardiff University, Cardiff, United Kingdom CF10 3AT

**Keywords:** infertility, Africa, quality of life, culture, gender, death

## Abstract

Despite the high prevalence of infertility within the sub-Saharan sterility belt, infertility in Zambia is understudied, particularly from a social perspective. Furthermore, few studies in sub-Saharan Africa include the infertility experiences of men. This article seeks to fill this gap by qualitatively describing the ways in which infertility in Zambia is socially and culturally loaded for both men and women. Demonstrating fertility is necessary to be considered a full adult, a real man or woman, and to leave a legacy after death. People in Zambia, including medical professionals, currently lack the necessary information and access to (or ability to provide) care to effectively resolve fertility issues. Infertile people manage their experience through a variety of social, emotional, spiritual, and medical strategies. However, no solution is considered adequate unless the intervention results in childbirth. In this way, infertility is about producing babies and the social meaning of that process, rather than the raising of children.

## Introduction

Zambia, a country in sub-Saharan Africa (SSA) of 13.1 million people (ZDHS 2014), experiences both high fertility and high infertility. The fertility rate is approximately 4.7 children/woman (ZDHS, 2018). Primary sterility rates, defined biologically as “never developing the capacity to reproduce,” are 15% for women (Sunil and Pillai, 2002) or higher. We currently do not have enough data to accurately estimate clinical infertility prevalence, though Zambia is in the SSA “infertility belt” where clinical infertility rates are significantly higher than global averages, partly due to reproductive tract infections and tubal occlusion (Inhorn and van Balen 2002). Infertility can create social problems for people in SSA because “having children is a self-evident part of the expected biography and not a matter of personal decision” (Hörbst, 2009); we add that children are also symbols of social status.

This paper uses the World Health Organization (WHO) definition of clinical infertility: “the failure to achieve a clinical pregnancy after 12 months or more of regular unprotected sexual intercourse” (WHO, 2020). Primary infertility refers to never having had a pregnancy that resulted in a live birth. Secondary infertility refers to difficulty conceiving or continuing a pregnancy to live birth after at least one pregnancy.

While infertility in SSA is stigmatised for both genders (Dyer, 2009), the social consequences for women are greater (Gerrits, 1997; Kimani and Olenja, 2001; Fledderjohann, 2012). Women experience increased domestic violence from husbands and in-laws (Ameh et al., 2007; Dhont, 2011; Stellar et al., 2016) and risk losing their identities when womanhood is defined through motherhood (Hollos, 2009; Silva, 2009).

In SSA, there are diverse beliefs about causes of infertility, including use of contraception and witchcraft (Dhont, 2011; Upton, 2001; Kimani and Olenja, 2001). Women are often blamed for causing their own infertility through “past promiscuity” (Kimani and Olenja, 2001), thereby inciting God’s wrath (Dutney, 2007). Childless women are often materially deprived by their families (Hollos, 2009). Infertility’s social significance in SSA leads to high demand for treatment (Gerrits, 2016; Ombelet, 2008, Inhorn and Patrizio, 2015). However, assisted reproductive technologies are usually financially and/ or logistically inaccessible in SSA (Gerrits, 2016; Stekelenburg et al., 2005; Gerrits and Shaw, 2010; Inhorn and Patrizio, 2015; Ombelet, 2008; Ndegwa, 2014; Nachtigall, 2006).

Despite its social importance, infertility is understudied in Zambia, as indicated by the lack of data on infertility rates. Two quantitative studies (Pantazis and Clark, 2014; Sunil and Pillai, 2002) have found high sterility prevalence and one study of the University Teaching Hospital (UTH) found correlation among infertility, age and history of pelvic procedure. Two qualitative studies have examined the social lives of elderly childless women in the Upper Zambezi region, including rural Zambia (Silva, 2009), and fear of infertility among young women in Lusaka Province (James, 2019). Both studies found that infertility deeply negatively impacts women’s social lives and status. Furthermore, few SSA studies have addressed the experiences of infertile men (Hörbst, 2009; Dyer et al., 2009). This paper begins to fill these gaps by describing how social and cultural meanings of infertility impact quality of life (QoL) for both men and women in Zambia.

Reproductive policy and research in Zambia focus on family planning, early pregnancy/marriage, and maternal health (Zambia Ministry of Health, 2018). Health policy acknowledges some barriers to infertility healthcare access but is hampered by a dearth of research on the extent, causes, and social impact of infertility in Zambia.

The findings presented are the first part of a mixed methods study of infertility in Zambia. We demonstrate that infertility is socially and culturally laden for both men and women. Lack of access to care and social responses negatively impact QoL for people with infertility. A second article will address knowledge produced about social meanings of infertility through mixed methods research and a novel approach to survey administration.

## Methods

The research described in this article was conducted by lead author Sydney Howe (SH) in Lusaka Province between June and September 2019 through a research collaboration involving Cardiff University, the University of Zambia (UNZA) and the University of Amsterdam; other publications from studies within the larger collaboration are under review or in preparation. Kabuswe Mbalazi (KM), a Master’s student and trained UNZA research assistant, translated approximately five interviews from Bemba or Nyanja, and transcribed most interviews with infertile participants. SH transcribed the remaining interviews, proofread, edited, and added to transcripts in consultation with KM, then coded all transcripts in Atlas.ti.

## Ethnography

Men and women of reproductive age who self- identified as infertile were recruited mostly through health workers in Lusaka and Chongwe, with assistance from UNZA academics and social contacts. Two interlocutors did not meet the WHO clinical standard because fertility histories were unknown before interviews. SH interviewed 22 infertile people (14 women, eight men) in 20 sessions (two married couples interviewed together). Six men came alone, which is unusual for an infertility study (Gerrits, 2018). All participants were Christian, most were lower or middle income, approximately half had a high school education or higher, and they were evenly divided between urban and rural areas. Most were married and all lived with their intimate partners. Nine had received biomedical infertility care. The age range was 21-39 (average 33).

SH also interviewed five doctors, five nurses, a pharmacist, a herbalist, several parents and Zambian academics, conducted participant-observation at community events and two gynaecology clinics, and contributed to community outreach projects about infertility. Narrative interviews covered topics related to infertility and QoL, including (but not limited to) personal relationships, work, death, living arrangements, medical care, religion and emotions. Interviews lasted between 30 minutes and 2.5 hours and were recorded with participant permission.

This study received ethical clearance from UNZA and the Zambian government. UNZA’s ethics committee requires reimbursement of participants for time and transportation; infertile participants in this study were paid 100 kwacha/person (~7 USD). Participants were given contact information and informed how their data would be used and of their right to withdraw from the study at any time. Consent was reaffirmed before questions about sex. No questions about having children outside the marriage were asked when couples interviewed together. All names and identifying details have been changed. In some cases, multiple pseudonyms were used if the details of someone’s story could expose their identity. Details were not changed for expert informants. All interviewees gave verbal informed consent for audio recording, except two gynaecologists, who agreed to written notes but not audio recording. Infertile participants signed reimbursement and written informed consent forms. On reimbursement records, the study’s title was changed to obscure the purpose of interviews, because participants were required to provide identifying details to receive money. By requiring submission of real names to a third party for payment, UNZA’s ethical requirements may have prevented complete anonymity.

## Results and discussion

### 

This section discusses ethnographic evidence for the impact of legacy, family, and solution-seeking on infertile people’s quality of life (QoL) through cases chosen from the cohort of participants. These cases were chosen because they clearly or demonstratively express concerns raised by many participants.

### Fertility, death and legacy

Social concerns about death and legacy factor heavily in discussions of infertility in Zambia, where people often die young and unexpectedly. Public health issues like HIV, tuberculosis, and cholera persist (Kapata et al., 2016; UNAIDS, 2019) but people also die because of accidents and health conditions that are treatable in wealthier countries. In three months, interlocutors and colleagues cancelled obligations nine times due to a death or hospitalization. Several participants had been widowed multiple times, though the oldest participant was 39. Children are considered the most effective way for families to connect with someone who has passed. As such, primary and secondary infertility impact people differently, especially because parenthood is locally defined as the ability to produce, not raise, children.

Nearly every interlocutor worried about death and legacy in relation to infertility. For some infertile participants, this worry manifested in concern about burial rites.

Let’s say if you are married, then you die, how they are going to bury that person is different from how they can bury someone who was married and has had kids...They will get maybe an old cob of maize and then put on the butt, and for those who have kids, they will just bury them normal. I remember an aunty...she had a friend who was old, she was about 50 years old and she never had child.... they would just tease and tell her, “Oh you don’t have any kids, so when you die, they are going to bury you like that.”(Melanie, Lusaka)

When they bury you, there will be no history. (Miranda, Lusaka)

Similarly to women in the Upper Zambezi, childless women in Lusaka demonstrate fear being forgotten by the community after death through their discussion of burial practices (Silva 2009). According to Lauren, an interlocutor from Lusaka who now lives in a rural area, burial rituals involving maize are designed to shame childless women even after death:

They get a maize cob and they, they, press it on... your anus...Meaning, that is your child....yeah (awkward laughter)...it’s like, shameful.

While this ritual is uncommon in Lusaka, the symbolism of maize and the intensity of burial rites discussions speak to the depth of concern that infertility raises about death in Zambia.

Many infertile people mentioned wanting to leave someone behind: Robert fantasised about raising a child with his father’s name so that “I would not have to lose the memory of him.” A child ensures the surviving wife has permanent ties to the extended family: childless widows in patrilocal marriages often lose rights to land in both their husband’s and their home villages (Chapato et al., 2011). Joy, a childless 22-year-old, recounted her panic during her husband’s malaria: “What if he dies, who will I be staying with?”

Because of high child mortality rates (UNICEF, 2018), one child is never considered enough. Nancy, who has a living daughter, but lost her son to sudden illness said, “They will just say you should have not only one child, at least you should have another one.” People sometimes used the term only to describe large families (i.e. “we are only six”), as though there could never be enough children. However, Lauren, who has a son, said, “I am not so worried because I know there is someone who carries my blood.” One child is still better than none.

Having a miscarriage sometimes provided protection from barrenness rumours and engendered sympathy for both men and women.

If you die without leaving any children, it is like... you vanish. But if you have a miscarriage, it is better, at least you are not barren. (Rachel, Lusaka).

To them, [if your wife miscarries,] then it means you are man enough and you can produce...They would say, “In Kafue you had girlfriends and we have never heard that you had a child there or whatever, or you made a girl pregnant and she aborted because she didn’t need the child”... Those are the words that those who mock me use. (Jacob, Chongwe)

In Nyanja, a dominant language in Lusaka, *sama bala* describes the condition of infertility for both genders. It means roughly “he (or she) does not produce (children)”. Even though the experience of infertility is gendered, production of children is not. Becoming (or making someone) pregnant publicly demonstrates that a person can produce children; thus, a miscarriage may improve social status. A lost pregnancy is often mourned like the death of an infant.

### Producing for the family

Because infertility raises questions of legacy, the extended family is implicated when a couple fails to conceive (Sewpaul, 1999); additionally, because early death is common, families have a stake in couples’ fertility decisions: “everyone takes over from you when you are incapacitated or when you die” (foster parent, Lusaka). Marriage across vast distances and different lineage systems is common. Matrilineality has been found to be somewhat protective for infertile women elsewhere in SSA (Gerrits, 2002), which may be true in Zambia too. Infertile women in matrilocal marriages generally reported higher QoL, particularly in rural areas, than women in patrilocal marriages ^1^. This is likely because the husband’s family usually puts pressure on the wife to conceive, and women sometimes received emotional support from nearby family members.

However, all childless men and women said they could not earn the respect traditionally accorded to parents. Many were not considered full adults by their families or communities. Systems of addressing one another, in both workplaces and families, explicitly indicate whether someone has children. In most Zambian cultures, parents are referred to as “Mother of (Child’s Name)” or “Father of (Child’s Name)” as a form of respect. Robert, who is childless, explained:

In our culture for you to earn respect is when you have a child. Even my younger siblings do have children so they are being much more respected than I am. Even decision-making, they would say: “Call Bashi Nanikani (Father of Nanikani).” It’s a meeting for parents. You are not a parent, so you are not eligible to be in that meeting. It’s like they are pulling you down, and your behaviour doesn’t change because you are like a bachelor...It’s quite a heavy load.

Whether naming traditions are particularly painful for childless people depends on their ethnic group’s tradition, sibling order, and the presence of same-gender siblings or cousins. For example, Amadeo said it hurts that, as the youngest son, his brother’s children will never call him “daddy”. However, Malaika noted that being called “mum” by a relative’s child is not the same as having her own:

“I would like someone who just [says] ‘Mum!’ and they’re mine.”

Infertility can cause people to lose or fail to obtain recognition as an adult man or woman.

I got my second marriage where I stayed for 7 years, I did not have a child and... [my husband said] I am not woman enough and cannot stay in the house, so that was how I was chased away again. (Lauren, Lusaka)

In this light, it is not surprising that the insults infertile people received were often gendered. Women in SSA are more likely than men to identify as infertile due to false beliefs about women’s responsibility for infertility (Hörbst, 2010). Several interlocutors who were neither infertile nor medical professionals equated male infertility with impotence and believed men who perform sexually cannot be infertile, a common assumption throughout SSA (James, 2019; Dhont et al., 2011; Araoye, 2003). In Chongwe, men without children may be mocked more than women; insults focus on their manhood. Peter’s friends called him *ngomwa*, which means both barren and impotent. They say he is not “man enough” to get someone pregnant, implying he cannot have sex at all. Mocking impacts men emotionally:

I feel so sad when they say a word like barren. I feel bad because most of my friends have children and some have got pregnancies... I ask myself questions that I cannot even answer myself... when they mock, it changes how I feel about it. (Jacob, Chongwe)

Some participants protected their spouses from mocking. Leroy’s wife does not rebut gossip that she takes birth control because she already has a child and he does not. Jacob protects his wife, who has fibroids, by telling others that either of them could be the problem. However, some men blamed their wives, despite internal doubts about their own fertility:

It hurts me a lot, and I complain and ask myself if I am the one with the problem, or it is her. But I have just made it like I am perfect and she has a problem. (Gregory, Chongwe)

Some infertile people, especially in small communities, continued to hang out with friends who mocked them to salvage long-term social connections. Some women found solace with other infertile women (Faria, 2018) either online or in person. Most participants receiving medical care were encouraged to go by friends or family. The chance to talk about infertility motivated several people to agree to an interview. No participants mentioned a mental health practitioner in response to questions about treatment and whether they confided in anyone. Several middle-class and most lower-class participants said SH was the first person who knew about their infertility experience other than family or doctors.

### Intimate partner violence

Several women in this study cited infertility as the reason their husbands neglected, beat and/or raped them. Intimate partner violence is widely accepted as normal or even a sign of love. Interlocutors casually referred to physical abuse and “being forced” to have sex, as though this was expected.

Stories of women being “chased from the house” (in which a woman is evicted by her husband and/ or his relatives, who bring in a new wife) were only reported in patrilocal marriages. One interlocutor, who lives in a rural area, was chased for failing to bear children, and now has one child with her current husband. Because one child is considered insufficient, her son does not protect her from being chased again if she fails to conceive. Her husband is now depriving her of food, saying, “food is for people who produce [children].”

Other projects within this collaboration also found that women feared being chased (James, 2019). However, the fear seems more prevalent than actual chasing incidents. Most participants had not personally experienced or witnessed either chasing or pregnancy-achieving cheating. Women likely deal with other forms of violence not covered in interviews; this is a topic for further research.

### Solution-seeking choices

#### Social solutions

Elsewhere in SSA, men often seek “social solutions” (Hörbst, 2009) to infertility by impregnating another woman or finding new wives. Men may do this to avoid paying for female-factor infertility treatment (Hörbst, 2009; Inhorn, 2012). However, in Zambia, few couples have access to treatment or testing. Therefore, both men and women often see divorce or infidelity as the only way for a man to prove his own fertility. Christian denomination did not impact infertile people’s consideration of divorce. In fact, several couples were active in different denominations and did not see this as a conflict in their marriages.

All men in this study either rejected the idea of fathering a child extramaritally, or felt emotionally conflicted about it:

I believe if a man, if he meets a woman, it’s final, until death. (Amadeo, Chongwe)

I feel bad because I know it would hurt her. (Gregory, Chongwe)

This conflict aligns with Inhorn’s (2012) findings in Egypt: despite societal ideas that infertility is an “inevitable source of marital distress and eventual divorce...many men’s and women’s stories suggest otherwise,” though barriers to adoption and ARTs exist in both countries ^2^. While fears of being chased or cheated on are clearly well-founded for women in Zambia, many men in this study avoided these social solutions.

#### Foster children

Fostering is extremely common in Zambia, parti- cularly among childless couples. In social terms, fostering does not make one a parent: the obligation to demonstrate fertility is not fulfilled by raising the children of others. Biological parenthood is integral to social identity; therefore, adoption is rare, despite relative legal accessibility, because it revokes the social status of any biological parent. Furthermore, many children are raised communally regardless of foster status. Participants discussed biological children, but never foster children, in infertility interviews unless specifically asked. Unsurprisingly, there are strong social norms around acceptable care practices for foster children:

I had an opportunity of a scholarship from a school for a child...Then I had no child to give that scholarship to, so I had to put my sister’s child on that scholarship. The family of my wife, I don’t know if they became jealous of what was happening, [they said], “Why would you be sponsoring another man’s child? Why don’t you have your own child?” It became like a confusion. It began maybe tempering with my wife’s brain, then she started running out on me, she began drinking. (Robert, Chongwe)

Robert tried to circumvent social rules within a matrilocal household for a child he loves. He maintains that caring for his foster son “counts” (makes him a father). However, the social consequences of treating a foster child like a biological son were enormous, eventually leading to separation from his wife.

#### Biomedical treatment

Accessible biomedical care, especially for non- life-threatening issues like infertility, is often not comprehensive or high-quality. Interestingly, all people who accessed any biomedical infertility care felt overwhelmingly positively about their treatment experience, though only one interlocutor had achieved pregnancy through treatment. Infertile people may feel they have no right to complain after receiving any treatment for a non-life-threatening problem.

However, many people avoid medical treatment, especially for sexually-coded (and thus, morally- charged) issues like STIs or abortion (including an infertile interlocutor married to a doctor). Even at public hospitals, the cost of a consultation (~50 kwacha) can be insurmountable ^3^, especially factoring in transportation costs. The problem often becomes much more serious, several Lusaka gynaecologists noted, by the time someone accesses care. Adding complication, outdated or medically-questionable practices can cause infections, making it even harder to get pregnant (Gerrits and Shaw, 2010).

Many people, including some healthcare providers, seem to have limited understanding of biological fertility. Three trainee nurses cited fear of infertility as the reason they personally refuse to use hormonal contraception. Several interlocutors in long-distance marriages had few days together each month. When together, they frequently had sex, up to seven times per day, hoping that more sex would help them conceive. Having so much sex without getting pregnant seemed to confirm participants’ fears, even though it is unlikely they had sex within the woman’s fertile window. Several participants may not have biomedical fertility problems, just poor timing.

Even with access to high-quality care, men may refuse diagnosis to avoid “proving” their infertility (Hörbst, 2010). To mitigate this, UTH and Lusaka IVF require women to bring their husbands for male-factor infertility testing, but many husbands refuse to go. Unlike UTH Gynaecology, Lusaka IVF, the only IVF clinic in Zambia, is highly visible as a fertility clinic, so there can be no pretence about why someone is there. The doctor asks men who refuse to come in for testing to send a semen sample with their wives.

At least one male interlocutor wanted to go for medical treatment, but his wife refused, to avoid diagnosis. However, traditional ideas about women’s responsibility for infertility and infertility as divine punishment generally prevent both genders from disclosing their situation or seeking help for (justified) fear of judgment, ridicule, and status or material loss.

#### Spirituality

Zambia identifies as a “Christian nation” (Constitution of Zambia, 1991); religion plays an enormous role in people’s lives. Most people attend church regularly; many spend their only day off in all-day services. Globally, people often draw “on religious as well as medical discourse to frame their experiences of and approaches to infertility” (Jennings, 2010). All infertile participants proclaimed their Christianity and saw children as a blessing from God. Many found comfort in prayer and the biblical story of Sarah giving birth in old age.

Like many from Abrahamic religious traditions, infertile people in Zambia often “experience infertility as a faith crisis: Why is God doing this? What have I done to deserve this from God?” (Dutney, 2007). This soul-searching happens at the community level too: women reported that others often looked for moral reasons for their infertility, including using birth control before marriage, past abortions, or acting “like a prostitute” (Prudence and Daniel, Chongwe). According to local health professionals, STIs and infections from unsafe abortions are common, resulting in a conflation of biomedicine and God’s will in the social meaning of infertility.

Generally, discussing faith seemed to lighten infertile people’s moods—they sat up straighter and their faces became more serene. For some, especially infertile women accused of un-Christian behaviour, being assumed to be a good Christian by an authority like a researcher may have been emotionally supportive.

Interactions between the church and traditional cultural practices are “slowly leading to the Christianization of traditional rituals” (Rasing, 1995). Christianity and traditional beliefs in urban and semi-rural Zambia are not mutually exclusive. Participants’ treatment narratives usually referenced spiritual forces, which they believed influenced treatment outcomes. Interactions with traditional healers (*ngangas*) are particularly revealing. Four infertile participants confirmed they had visited a traditional healer for infertility. They said they were given herbs or plants to ingest or carry with them, rather than spells. However, more participants have likely used a *nganga*’s services (including spells), because seeing a *nganga* is stigmatised; clients risk accusations of practicing witchcraft by seeking services. Which services are acceptable appears to be quite personal: some refuse to visit a *nganga* “because I am a Christian,” while others see no conflict, and a third group would only use medicine (herbs) not spells. However, witchcraft is part of everyday life in Zambia and some participants feared someone had cast a spell that made them infertile.

The biomedical and the spiritual are often entangled. Traditional healers address both medical (i.e. hormone imbalances) and spiritual (i.e. evil relatives) fertility problems. Furthermore, biomedical problems are often attributed to spiritual causes. For example, an interlocutor ascribed a post- C-Section infection to “the devil,” not biomedical failures. Outside Zambia, infertile people justify fertility treatment outcomes as “God’s will” (Jennings, 2010). However, participants in Zambia seemed to see Christian belief as the solution and biomedicine as the possible miracle, rather than the reverse. One participant talked herself out of “leaving it up to God” to fully engage with biomedicine: “I understand these things are science... I do pray for a baby, but I understand most of the work has be done by me.”

## Conclusion

Infertility in Zambia remains socially taboo and under-researched, with few biomedical treatment options available. Conceptualisations of death and legacy, family and gender, and biomedicine and religion all contribute to the meanings of infertility. Infertility challenges people’s understandings of their purpose in life, their identity and relationships, and their faith in both God and medicine. The experience of infertility negatively impacts quality of life by disrupting infertile people’s core beliefs about their identity and place in their community. Social structures, local understandings of infertility, and traditions can exacerbate an already-difficult period for infertile, and particularly childless, men and women. Infertility affects both genders but differently: men often experience more mocking, and women may experience significant material, physical, and emotional abuse.

Due to the COVID-19 pandemic, examining the meanings of infertility in relation to death has acquired new importance. The constant presence of death in daily life in Zambia makes legacy a primary concern. Children are often the only way avoid “leaving nothing behind” (Melanie, Lusaka). Expecting death changes how people approach medical care: care for the soul is often given priority over care of the body. These meanings could become stronger in Zambia during the pandemic, and in other countries with high COVID-19 death tolls.

## Limitations

This study is biased towards infertile people seeking medical treatment. The women interviewed who were in treatment; all had husbands who had been tested. All men interviewed desired medical treatment. Infertile people participating in research may be more willing to take social risks to resolve fertility problems, may have more supportive partners, and/or may be less influenced by cultural notions of women’s responsibility for infertility and gender roles (Gerrits, 2018). Further research is necessary to understand experiences beyond this limited cohort. Interruptions prevented complete privacy during interviews, even in closed rooms or behind curtains in patient consultation areas at UTH. However, no participants seemed uncomfortable unless interviewed with their spouse (Gerrits, 2018). Nonetheless, lack of complete privacy likely impacted some responses.

## Policy and recommendation

The Deans of Research and Medicine at UNZA and Heads of Obstetrics and Gynaecology at UTH required Zambia-specific data to support (and justify) investment in a Ministry of Health-based infertility research group; this study contributes QoL data for infertile people. A forthcoming policy brief informed by our findings discusses how and whether to develop this initiative. Infertility prevalence represents a particularly important area for further research.

Basic infertility education would improve understanding of biological fertility among lay people and professionals and dispel myths. Curricula should include information about who can be infertile, biomedical causes of infertility, and acknowledgement of infertile people’s social/emotional suffering. Effective education can be spontaneous and informal. During an improvised infertility play informed by this study produced by local NGO Kingdom Culture in a Lusaka township, the audience gasped audibly when the actors revealed the husband was infertile; several people immediately asked the director if this was possible. He referred them to UTH: they had not realised infertility can be treated medically.

Other than increasing healthcare access, QoL for infertile people may be improved by reframing public discussions of fostering. Recognising foster and biological parenthood as equally important could give infertile couples some social relief and neglected children more secure homes.

Finally, stigma cannot be broken in silence. Healthcare policies and education should increase discussion of infertility without shaming infertile people. Men may have more power in their communities; their experiences must be included. Enforcing domestic violence laws and property rights would ease infertility consequences that affect women disproportionately.

## Footnotes

^1^ Some interlocutors in matrilineal communities lived near the husband’s family (patrilocal marriage), or the reverse.

^2^ Social/financial in Zambia, legal in Egypt .

^3^ 50 Zambian kwacha ≈ 3.50 USD. Minimum wage ≈30 USD/ month. A living wage is 184 USD (Koyi 2019). Many interlocutors were unemployed or worked informally for less than minimum wage. 60% of people in Zambia cannot meet basic needs (ZDHS 2014).

## References

[1] Agadjanian V (2005). Fraught with Ambivalence: Reproductive Intentions and Contraceptive Choices in a Sub-Saharan Fertility Transition.. Popul Res Policy Rev.

[2] Ameh N, Kene TS, Onuh SO (2008). Burden of domestic violence amongst infertile women attending infertility clinics in Nigeria.. Nig J Med.

[3] Baylies C (2000). The impact of HIV on family size preference in Zambia.. Reprod Health Matters.

[4] Bennett LR, de Kok B (2018). Reproductive Desires and Disappointments.. Med Anthropol.

[5] Boivin J, Takefman J, Braverman A (2011). The Fertility Quality of Life (FertiQoL) tool: development and general psychometric properties.. Fertil Steril.

[6] Bos H, van Balen F (2005). Social and cultural factors in infertility and childlessness. Patient Educ Couns.

[7] Chapman K, Gordon G (1999). Gend Dev. Reproductive health technologies and gender: Is participation the key.

[8] Chapoto A, Jayne TS, Mason NM (2011). Widows’ land security in the era of HIV/AIDS: panel survey evidence from Zambia.. Econ Dev Cult Change.

[9] Chola M, Michelo C (2016). Proximate determinants of fertility in Zambia: Analysis of the 2007 Zambia Demographic and Health Survey.. Int J Popul Res.

[10] Corbin J, Morse JM (2003). The unstructured interactive interview: issues of reciprocity and risks when dealing with sensitive topics.. Qual Inq.

[11] Dhont N, van de Wijgert J, Coene G (2011). “Mama and papa nothing”: living with infertility among an urban population in Kigali, Rwanda.. Hum Reprod.

[12] Dutney A (2007). Religion, infertility and assisted reproductive technology.. Best Pract Res Cl Ob.

[13] Dyer SJ, Vinoos L, Ataguba JE (2017). Poor recovery of households from out-of-pocket payment for assisted reproductive technology.. Hum Reprod.

[14] Dyer S, Lombard C, Van der Spuy Z (2009). Psychological distress among men suffering from couple infertility in South Africa: a quantitative assessment.. Hum Reprod.

[15] Faria I (2018). Therapeutic navigations and social networking: Mozambican women’s quests for fertility.. Med Anthropol.

[16] Fledderjohann J, Barnes LW (2018). Reimagining infertility: a critical examination of fertility norms, geopolitics and survey bias.. Health Policy Plan.

[17] Gerrits T (1997). Social and cultural aspects of infertility in Mozambique.. Patient Educ Couns.

[18] Gerrits T, Shaw M (2010). Biomedical infertility care in sub-Saharan Africa: a social science - review of current practices, experiences and viewpoints.. Facts Views Vis Obgyn.

[19] Gerrits T (2012). Biomedical infertility care in low resource countries: barriers and access: introduction.. Facts Views Vis Obgyn.

[20] Gerrits T (2018). Gender dynamics, sensitive issues and ethical considerations in “joint interviews” with Dutch couples undergoing fertility treatments.. Salut e Soc.

[21] Gerrits T Patient-centred IVF: bioethics and care in a Dutch clinic New York: Berghahn Books; 2016 10.1080/20502877.2018.1525653 30244667

[22] Greil AL, Shreffler KM, Johnson KM (2013). The Importance of Social Cues for Discretionary Health Services Utilization: The Case of Infertility.. Sociol Inq.

[23] Hammarberg K, Trounson A, McBain J (2018). Improving access to ART in low-income settings through knowledge transfer: a case study from Zimbabwe.. Hum Reprod Open.

[24] Hollos M, Larsen U, Obono O (2009). The problem of infertility in high fertility populations: meanings, consequences and coping mechanisms in two Nigerian communities.. Soc Sci Med.

[25] Hörbst V (2009). In the making: assisted reproductive technologies in Mali, West Africa.. Anthropology News.

[26] Hörbst V (2010). Male perspectives on infertility and assisted reproductive technologies (ART) in sub-Saharan contexts.. Facts Views Vis Obgyn.

[27] Hörbst V, Wolf A (2014). ARVs and ARTs: Medicoscapes and the unequal place-making for biomedical treatments in sub-Saharan Africa: ARVs and ARTs.. Med Anthropol Q.

[28] Inhorn MC, Patrizio P (2015). Infertility around the globe: new thinking on gender, reproductive technologies and global movements in the 21st century.. Hum Reprod Update.

[29] Inhorn MC The new Arab man: emergent masculinities, technologies, and Islam in the Middle East. Princeton, NJ: Princeton University Press; 2012

[30] Inhorn MC, van Balen F Infertility around the globe: new thinking on childlessness, gender, and reproductive technologies Berkeley: University of California Press; 2002

[31] Jaffré Y, Suh S (2016). Where the lay and the technical meet: using an anthropology of interfaces to explain persistent reproductive health disparities in West Africa.. Soc Sci Med.

[32] James K “I am just a tree without fruits”: Understanding fear of infertility among young women in Zambia through metaphor-based art. Anthropology University of Amsterdam 2019

[33] Jennings PK (2010). “God had something else in mind”: Family, religion, and infertility.. J Contemp Ethnogr.

[34] Kalima-Munalula M, Ahmed Y, Vwalika B (2017). Factors associated with infertility among women attending the gynaecology clinic at University Teaching Hospital, Lusaka, Zambia.. Med J Zambia.

[35] Kapata N, Chanda-Kapata P, Ngosa W (2016). The prevalence of tuberculosis in Zambia: results from the first national TB prevalence survey, 2013–2014.. PLoS ONE.

[36] Kavanaugh ML, Moore AM, Akinyemi O (2013). Community attitudes towards childbearing and abortion among HIV-positive women in Nigeria and Zambia.. Culture, health & sexuality.

[37] Kimani V, Olenja J (2001). Infertility: Cultural dimensions and impact on women in selected communities in Kenya.. Afr Anthropol.

[38] Koyi G Wage Indicator 2019. Minimum and living wages in Zambia: some analytical considerations for improving workers’ conditions. Africa Labor Research. Education Institute; 2019

[39] Mariano EC (2004). Involuntary childlessness among the Shangana (Mozambique).. J Reprod Infant Psychol.

[40] Moore AM, Keogh S, Kavanaugh M (2014). Bucking social norms: examining anomalous fertility aspirations in the face of HIV in Lusaka, Zambia.. Soc Sci Med.

[41] Mugweni E, Pearson S, Omar M (2012). Traditional gender roles, forced sex and HIV in Zimbabwean marriages.. Cult Health Sex.

[42] Nachtigall RD (2006). International disparities in access to infertility services.. Fertil Steril.

[43] Ndegwa SW (2016). Affordable ART in Kenya: The only hope for involuntary childlessness.. Facts Views Vis Obgyn.

[44] Ombelet W (2008). False perceptions and common misunderstandings surrounding the subject of infertility in developing countries.. ESHRE Monographs.

[45] Ombelet W (2014). Is global access to infertility care realistic? The Walking Egg Project.. Reprod Biomed Online.

[46] Pantazis A, Clark SJ (2014). Male and female sterility in Zambia.. Dem Res.

[47] Rasing T The bush burnt, the stones remain: female initiation rites in urban Zambia Hamburg: Lit; 2001

[48] Sewpaul V (1999). Culture, religion, and infertility: a South African perspective.. Br J Soc Work.

[49] Silva S (2009). Mothers of solitude: childlessness and intersubjectivity in the upper Zambezi.. Anthropol Humanism.

[50] Spronk R (2014). The idea of African men: dealing with the cultural contradictions of sex in academia and in Kenya.. Cult Health Sex.

[51] Stekelenburg J, Jager BE, Kolk PR (2005). Health care seeking behaviour and utilisation of traditional healers in Kalabo, Zambia.. Health Policy.

[52] Stellar C, Garcia-Moreno C, Temmerman M (2016). A systematic review and narrative report of the relationship between infertility, subfertility, and intimate partner violence.. Int J Gynecol Obstet.

[53] Sunil TS, Pillai VK (2002). Sterility in Zambia.. Ann Hum Biol.

[54] UNAIDS Country Report - Zambia [Internet] UNAIDS; 2019 Available from: https://www.unaids.org/en/regionscountries/countries/zambia

[55] Upton RL (2001). “Infertility makes you invisible”: Gender, health and the negotiation of fertility in northern Botswana.. J South Afr Stud.

[56] van Balen F, Bos HMW (2009). The social and cultural consequences of being childless in poor-resource areas.. Facts Views Vis Obgyn.

[57] (2020). Multiple definitions of infertility. Sexual and reproductive health..

[58] Zambia The Constitution of Zambia. 1991

[59] UNFPA RMNCAH-N Communication and Advocacy Strategy 2018-2021 Zambia Ministry of Health; 2018

[60] (2015). Zambia Demographic and Health Survey 2013-14.

[61] Zambia Demographic and Health Survey. Lusaka, Zambia: Zambia Statistics Agency; Ministry of Health; University eaching Hospital Virology Laboratory; 2019

